# Self-Assembled Peptide Hydrogels in Regenerative Medicine

**DOI:** 10.3390/gels9080653

**Published:** 2023-08-14

**Authors:** Shuangyang Li, Qixuan Yu, Hongpeng Li, Meiqi Chen, Ye Jin, Da Liu

**Affiliations:** 1 School of Pharmacy, Changchun University of Chinese Medicine, Changchun 130117, China; lisyang1206@163.com (S.L.); yuqx0221@163.com (Q.Y.); lhp990504@163.com (H.L.); 15567958349@163.com (M.C.); 2 Northeast Asia Research Institute of Traditional Chinese Medicine, Changchun University of Chinese Medicine, Changchun 130117, China

**Keywords:** self-assembling peptide hydrogels, skin regeneration, bone regeneration, nerve regeneration, self-assembling peptide

## Abstract

Regenerative medicine is a complex discipline that is becoming a hot research topic. Skin, bone, and nerve regeneration dominate current treatments in regenerative medicine. A new type of drug is urgently needed for their treatment due to their high vulnerability to damage and weak self-repairing ability. A self-assembled peptide hydrogel is a good scaffolding material in regenerative medicine because it is similar to the cytoplasmic matrix environment; it promotes cell adhesion, migration, proliferation, and division; and its degradation products are natural and harmless proteins. However, fewer studies have examined the specific mechanisms of self-assembled peptide hydrogels in promoting tissue regeneration. This review summarizes the applications and mechanisms of self-assembled short peptide and peptide hydrogels in skin, bone, and neural healing to improve their applications in tissue healing and regeneration.

## 1. Introduction

The term regenerative medicine was first coined in 600 BC. The first surgical textbook in history records that an Indian physician described how to repair torn earlobes with skin from the cheeks and reconstruct a nose with a forehead flap, which can be regarded as the beginning of regenerative medicine in human history [1,2,3]. Regenerative medicine is a new direction in the development of modern medicine. Regenerative medicine seeks to use the theoretical methods of biology and engineering to create lost or functionally impaired tissues and organs such that they have the structure and function of normal tissues and organs [4]. Treating diseases, such as skin defects and severe bone damage, in patients with diabetes faces many challenges due to the weak self-repairing and regenerative capacity of organs, such as skin and bone, and patients suffer a great deal of pain during treatment [5,6]. Therefore, searching for therapeutic strategies that rapidly repair damaged tissues and shorten patients’ disease duration are of great clinical and social significance [7]. If regenerative medicine is used to treat these diseases, it will maximize patients’ quality of life and play an indispensable role in advancing medicine [8]. The selection of biomaterial scaffolds is a critical part of regenerative medicine, and traditional scaffold materials have certain defects, such as poor bioavailability, that affect their release rate of drugs for specific purposes [9].

A self-assembled hydrogel is a good biological scaffold. It has roles in cell attachment and migration and the ability to promote cell–cell interactions, cell proliferation, and cell division [10,11]. Therefore, it can improve biocompatibility and control the biodegradation of drugs [12]. Some organic small molecules can be at very low concentrations (mass fraction < 1%) in the water through the molecular self-assembly process to form a supramolecular network structure that will be wrapped in the water to form a non-flowing colloidal structure called a hydrogel [13]. Most currently studied gel delivery system matrices are created from gelatin, alginate, hyaluronic acid, chondroitin sulfate, polyethylene glycol, and poly(vinyl alcohol) [14]. Among them, self-assembling peptide (SAP) materials can rely on physical non-covalent bonding to self-assemble to form nanofibrous hydrogels, which can provide cells with a three-dimensional (3D) microenvironment similar to that of the natural extracellular matrix (ECM), and affect their biological behavior, such as growth, migration, and differentiation [15,16].

Unlike conventional materials, SAP molecules assemble into stable structures through various non-covalent interactions that form from molecules to nanofibers to the final hydrogel network, often exhibiting a hierarchical microstructure [17]. In addition, the structures of SAP hydrogels are similar to that of the ECM, and their degradation rate is compatible with the growth rate of many tissues, resulting in them being ideal scaffolds for tissue engineering. SAP hydrogels are primarily categorized into SAP and self-assembled short peptide (SASP) hydrogels [18]. Many factors affect the SAP hydrogels, including pH [19], peptide concentration [20], ion concentration [21], temperature [22], and chirality [23] (Table 1).

Therefore, using SAP hydrogels in regenerative medicine is of great significance. In this article, we mainly review the progress of SAP and SASP hydrogels loaded with different drugs in tissue regeneration, intending to contribute to studying SAP hydrogels and advancing regenerative medicine.

## 2. Application of SASP Hydrogels in Regenerative Medicine

SASP hydrogels comprise alternating hydrophilic and hydrophobic amino acid sequences that are stimulated under certain conditions (e.g., ionic concentration, temperature, and pH) to form a stable β-folded structure spontaneously and regularly through non-covalent bonding [26]. These structures are stacked into nanofibers to form an interwoven matrix, forming a hydrogel scaffold with >99% water content [27]. Such hydrogels can be used as good scaffold materials in regenerative medicine because they have a suitable pore size to promote cell proliferation and migration, and their degradation products are amino acids [28]. As a new type of biomaterial, SASP hydrogels have advantages such as programmability, low cost, good viscoelasticity, high biocompatibility, and low immunogenicity, and have been widely used in the medical field for tissue engineering, drug release, hemostasis, and antimicrobial action [29].

### 2.1. Application of SASP Hydrogels in Skin Regeneration

As the body’s largest organ in physical contact with the outside world, preventing the entry of foreign objects and pathogens and the loss of body fluids, amongst other functions, the skin is the body’s first direct line of defense against external damage. However, the skin’s repair capacity is poor due to its continual exposure to the external world, resulting in it having difficulty healing, with a long recovery cycle with traditional treatment, during which the patient will suffer much pain [30,31]. For example, according to an analysis of a large number of scientific research data reported by American scientists, the United States now spends > 1 billion dollars annually on chronic, intractable wounds, representing ~2% of the total economy of Europe [32,33]. While autologous skin grafts are often used in clinical practice, especially for large skin defects, the lack of donor sources and secondary injuries limit their application. Moreover, allogeneic grafts face immune rejection, resulting in it being hard to achieve better results with them [34]. Therefore, developing novel therapeutic approaches is critical, and applying SASP hydrogels as a novel agent for skin rejuvenation has considerable potential [35].

Wound healing is a long-term, multi-stage biological process that includes hemostasis, inflammation, proliferation, and tissue remodeling, requiring specific novel therapeutic tools to provide comprehensive and convenient treatment [36]. When the skin is injured, bleeding and microbial invasion are inescapable, leading to wound infection, disrupting the skin’s normal self-healing mechanism, and endangering human life [37]. Feng et al. created a hydrogel by coupling the SAP RADA16 with an antimicrobial peptide (AMP) and preparing it by adding poly(N-isopropylacrylamide) containing the mechano growth factor (MGF) E peptide, which was subsequently shown by rat whole skin experiments to significantly accelerate wound healing by accelerating epithelialization, neovascularization, and promoting collagen fibrillar production compared to commercial dressings. This study demonstrated that SASP hydrogels have good antimicrobial and hemostatic efficacy and play a vital role in the first stage of wound healing [17]. In addition, Lei et al. reported the first preparation of tannic acid (TA)-short interfering RNA (siRNA) nanogels based on the self-assembly interactions of TA and siRNA. They found that combining electrostimulation and TA-siRNA hydrogel treatment accelerated the healing of diabetic wounds by decreasing the levels of reactive oxygen species and matrix metallopeptidase 9 (MMP9) and promoting macrophage polarization, collagen production, and angiogenesis for the anti-inflammatory phase of the second wound healing stage and the remodeling phase of the third [38]. Lou et al. found that hydrogel was retained in wounds for seven days after injection into a diabetic mouse model, accelerating wound healing by ~20% compared to controls and promoting angiogenesis (e.g., vascular endothelial growth factor A [VEGFA]^+^ and platelet and endothelial cell adhesion molecule 1 [PECAM1/CD31]^+^ capillaries), cell proliferation (e.g., proliferating cell nuclear antigen [PCNA]+ cells), granulation tissue formation (e.g., alpha-smooth muscle actin [α-SMA]), and ECM deposition/modeling (e.g., type I collagen and fibronectin). In addition, the KGH hydrogel created a 3D microenvironment for skin cells in vitro that maintained the continuous growth of cell spheroids and increased the secretion of ECM proteins (e.g., lamellipodial adhesion proteins) and growth factors (e.g., platelet-derived growth factor subunit B [PDGFB], VEGFA, and transforming growth factor-beta [TGF-β]) by keratinocyte-forming cells in the skin, as compared to conventional dosage forms. Their results showed that the SAP hydrogel (KGH) could enhance ECM remodeling and angiogenesis, thereby promoting chronic wound healing in diabetic mice, and mechanistically, it may promote wound tissue regeneration by activating the Rho/Rho-associated coiled-coil containing protein kinase (Rho/ROCK) and TGF-β/mitogen-activated protein kinase (MEK)/mitogen-activated protein kinase (MAPK) pathways [39]. Lu et al. similarly explored using the SAP SCIBIOIII to form a special nanofiber structure mimicking the natural ECM in chronic diabetic wound repair. They showed that the SCIBIOIII hydrogel had good in vitro biocompatibility and could create a 3D culture microenvironment for continuous spherical skin cell growth [40]. In addition, the SCIBIOIII hydrogel significantly improved wound closure, collagen deposition, and tissue remodeling and enhanced chronic wound angiogenesis in diabetic mice.

**Figure 1:**
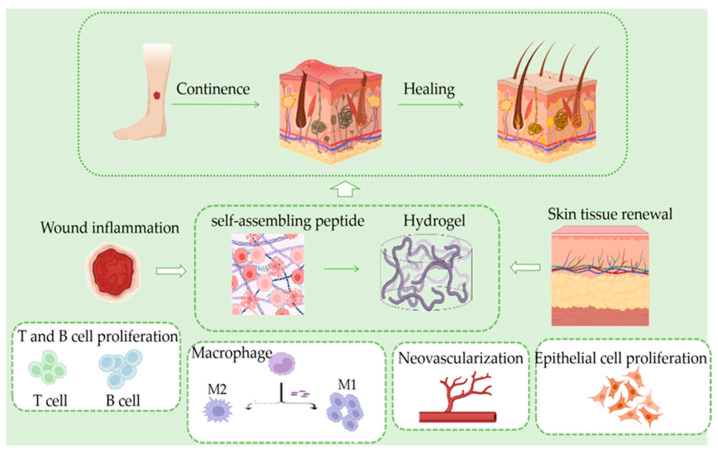
In the skin tissue regeneration process; inflammation occurs after hemostasis. At this stage, T and B cells proliferate rapidly, and macrophages polarize from M1 to M2 type, which synergistically inhibit inflammation, followed by the generation of neovascularization and granulation tissues in the skin, resulting in new skin.

The SASP hydrogel has the excellent properties of traditional hydrogels but also has the advantages of drug transport, protein degradation products without toxic side effects on the human body, and using short peptides as raw materials, which is itself a kind of protein, for the final water-based hydrogel, ensuring its safety. Skin regeneration is a research hot spot and a complex area of regenerative medicine. SASPs in skin regeneration have broad applications, and increasing numbers of researchers are investing in this research (Figure 1).

### 2.2. Application of SASP Hydrogels in the Field of Bone Regeneration

As an essential part of the body, bone tissue shows difficulty recovering from injuries and has a complex recovery process due to its recovery being affected by many factors. Therefore, the problem of bone regeneration has aroused a great deal of interest from researchers, and SASP hydrogel scaffolds have become a powerful alternative solution to the current tissue regeneration strategy [41,42]. As an alternative, SASPs have high resistance to pH or temperature changes due to the precise control of their chemical composition [43]. Hydrogels can be divided into two categories according to their raw materials: natural and synthetic. The instability and poor mechanical strength of natural hydrogels mean they cannot provide a microenvironment similar to that of cytoplasmic matrices, limiting their application in bone regeneration [44]. Therefore, there is an urgent need for a novel material to overcome this problem, and SAP hydrogels are an excellent choice with considerable potential for bone tissue regeneration applications.

Current treatments for bone defects include repairing soft tissues and reconstructing bone connections. However, the risk of recurrence due to the inability to accurately remove the infected foci leads to treatment failure [45]. In this context, filling bone defects with hydrogel is important in triggering bone healing and providing structural and mechanical support during this process [46]. One study examined scaffolds comprised of SASP KLD (KLDLKLDLKLDL) by direct coupling of short bioactive motifs O1 (EEGGC) and O2 (EEEEE), whose bioactivity has been demonstrated for osteogenic differentiation. They self-assembled them at different concentrations (0.5%, 1%, and 2%) and used atomic force microscopy and scanning electron microscopy to observe their molecular integration. Real-time polymerase chain reaction and immunofluorescence analyses showed significantly increased expression levels of key osteogenic markers of alkaline phosphatase (ALP) activity, collagen type I (COL-1), osteoblastin (OP), and osteocalcin (cyanate) due to the incorporation of glutamate residues into the KLD peptide. A series of experiments with this self-assembled SASP hydrogel also showed significant promotion of bone regeneration. The KLD SAP was functionalized by extending its amino terminus with a biomimetic glutamate template peptide (EEEEE) using direct solid-phase synthesis. The glutamate template peptide increased the hydrophilicity of the KLD SAP, successfully forming an SAP hydrogel. Further studies showed that glutamate residues were the major fragments affecting the bone ECM’s non-collagen mineralization and osteogenic differentiation. These results showed that the KLD SAP functionalized with the EEEEE template peptide can potentially induce osteogenesis. Several studies have highlighted the importance of improving the bioactivity of synthetic SAP hydrogels for better bone tissue regeneration. It is believed that this finding will help to optimize biofunctionalized biomaterials further [47]. Lv et al. designed a novel functionalized SAP hydrogel (KLD-12/KLD-12-LPP, KLPP) containing link protein N-terminal peptide (LPP) to optimize cartilage repair, and characterized the KLPP hydrogel using transmission electron microscopy and rheometry. Their subsequent experiments showed that KLPP, a simple injectable functionalized SAP hydrogel, promoted the simultaneous recruitment of endogenous chondrocytes and bone marrow mesenchymal stem cells (BMSCs), facilitated interfacial integration, and promoted cartilage regeneration, showing great potential as a one-step surgical strategy for endogenous cartilage repair [48].

Saha et al. hypothesized that combining an oligopeptide P11-4 hydrogel with human dental pulp stromal cells could promote bone regeneration in a rat cranial defect model in vivo. Their experimental results showed that the pH-sensitive self-assembled β-peptides could reversibly switch between fluid and gel phases in response to environmental triggers, resulting in them being potentially useful injectable scaffolds for skeletal tissue engineering applications. The ability of SAPs to self-assemble when triggered by environmental cues and their potential to modulate cell adhesion, mechanical stiffness, and biodegradation by altering peptide sequences result in them being a cellular tissue repair system [49]. In addition, the ion-complementary short peptide FEFEFKFK designed by Wang et al. could self-assemble into β-folded nanofibers in solution and eventually form hydrogels. SAP hydrogels combine the advantages of natural and synthetic biomaterials while overcoming their drawbacks. The peptide sequences can be easily modified to control the final mechanical and structural properties. SAP hydrogels may show good biocompatibility because their breakdown products will be identical to proteins synthesized in vivo. Bovine articular chondrocytes encapsulated within FEFEFK feather keratin hydrogels could proliferate and produce type II collagen, a component of the cartilage ECM, and had a rounded morphology, suggesting that the cells retained their phenotype for treating degenerative disk disease and lower back pain [50].

Currently, bone defects have been widely repaired in clinical practice. However, both autologous and allogeneic grafts have certain limitations, and artificial bone suffers from poor biocompatibility and other problems. Therefore, bone tissue engineering technology is expected to become a new therapeutic tool. SASP hydrogels are emerging biomaterials widely used in biomedical applications due to their programmability, low cost, good biocompatibility, and non-toxic degradation products. However, their poor stability, biocompatibility, and mechanical properties mean their degradation rate fails to keep pace with tissue growth, leading to the early release of seed cells, resulting in them failing to reach their intended location and a tendency to cause inflammation. Its weaker mechanical strength limits its ability to be applied to relatively rigid tissues, such as cartilage and bone tissues, which is its main problem in clinical bone tissue engineering applications (Table 2).

### 2.3. SASP Hydrogels in the Field of Nerve Regeneration

Neural regeneration, also known as “neural redifferentiation,” is the process in which neurons or glial cells are wholly or partially replaced or repaired at the injury site, restoring their normal function. This regeneration may occur by producing new neurons, glia, axons, myelin sheaths, or synapses [52,53,54]. Neurological injuries affect >90,000 individuals annually. It is estimated that spinal cord injuries alone affect 10,000 individuals annually. The high injury rate and difficulty recovering from neurological injuries create an urgent need for new types of material scaffolds to address this issue [55]. Currently, hydrogels are widely used in tissue engineering as a flexible material, and some hydrogels designed from natural biomaterials have many defects [56]. They must be modified to have better physicochemical properties. However, some chemical cross-linking agents and other substances introduced in the modification process are unsuitable for this purpose [57], potentially reducing their biocompatibility. In order to provide both good mechanical properties and biocompatibility, many studies have proposed the design of molecular materials based on SASPs and their derivatives [58].

Autologous nerve grafts are the current gold standard of treatment for long segmental nerve deficits. However, the lack of donor tissue and nerves means they can cause secondary neuromas and painful peripheral neuropathies, resulting in suboptimal prognoses for patients [59]. Therefore, many studies have combined nerve regeneration with SASP hydrogels to provide a new direction for advancing regenerative medicine [60]. Cheng et al. attached the laminin-derived peptide IKVAV to the end of RADA16 to obtain a functionalized β-folded SASP. The created RADA16-IKVAV hydrogel scaffolds had similar mechanical strength to brain tissues, potentially enhancing the adhesion of neural stem cells and their differentiation into neuronal cells [51]. RADA(16)-IKVAV could self-assemble into a nanofibrous morphology with a bilayer β-sheet structure and become a gel with mechanical stiffness similar to brain tissue to promote neural tissue regeneration. RADA16-I (Ac-[RADA]4-CONH2) is a widely studied SASP that forms a stable secondary structure through ordered self-assembly, forming nanofiber hydrogels. By attaching functional short peptide sequences to the end of RADA16-I, the modified short peptide can acquire further biological functions, expanding its application scope. One study created SASP hydrogels by diluting RADA16-I with Milli-Q water and autoclaving to create autoclaved microtubules. They then added Dulbecco’s modified eagle medium and conducted experiments on a rat model of vocal cord injury. Their results showed that the number of neurofilament-positive areas and myelinated nerves was significantly higher in the RADA16-I (+) group than in the RADA16-I (−) group, indicating the effectiveness of RADA16-I on nerve tissue regeneration [61]. The SASP RADA-16-I-BMPH1 has shown positive results in neural stem cell culture and spinal cord regeneration. Studies have successfully prepared hybrid-aligned poly(lactic-co-glycolic acid) (PLGA) nanofiber scaffolds with an SAP (RADA16-IBMHP1) by surface coating and co-electrostatic spinning [62]. Compared with the unmodified PLGA scaffolds, rat cevocytes showed enhanced adhesion to peptide-coated and co-spun silk scaffolds with typical bipolar morphology and extensibility. There was a more uniform distribution of peptides with rod-like structures on peptide co-spun PLGA nanofiber surface-coated scaffolds. Gene expression patterns for ceovanocyte markers, such as peripheral myelin protein 22 (PMP22), neural cell adhesion molecule 1 (NCAM1), and glial fibrillary acidic protein (GFAP), indicate that these scaffolds could repair peripheral nerve deficits by enhancing ax.onal regeneration and functional nerve restoration [63].

While SASPs have been shown to promote neural tissue regeneration, research in this area is still in its infancy, and most systematic studies have focused on in vitro cell culture and in vivo combined stem cell transplantation for more than a decade [64]. The SASP material has a similar elastic modulus to neural tissue and can better mimic the ECM environment of neural tissue [65,66]. However, most current research focuses on joint stem cell repair of spinal cord injury, and the research on neurological diseases is not comprehensive and in-depth enough [67]. It is believed that applying SASPs in tissue engineering will reach new heights with the continuous optimization of the types and properties of these peptides.

## 3. SAP Hydrogels

Some polypeptide sequences can self-assemble to form supramolecular structures under intra- and inter-molecular forces. Peptide-based materials can respond to the physiological environment by self-assembling to form microstructures with functionality for trauma repair [10]. SAP hydrogels are naturally biocompatible and degradable with ligand–receptor recognition, stimulus-responsive self-assembly, and mimic ECMs [24]. Self-assembly of peptides is an effective way to prepare nanostructured materials, and the gelation of peptides is a dynamic process that can cause aggregation through its interactions. SAP hydrogels have a wide range of applications in nanotechnology and biomedicine, including local drug delivery [68]. SAP hydrogels can be simply prepared to carry drugs, cytokines, or cells to a specific site, and are broken down into biologically active peptide sequences or natural amino acids that can be used to repair the surrounding tissue [69]. The molecule’s symmetry is also vital when designing SAPs, and many self-assembling molecules are symmetrical because such molecules require fewer types of contact interfaces and are more likely to form aggregates during assembly interactions [70].

### 3.1. SAP Hydrogels in Bone Regeneration

SAP hydrogels can bionically mimic the ECM and play a facilitating role in cell adhesion and migration. They also have low immunogenicity and suitable mechanical properties and have shown good osteogenic properties in vivo and in vitro [71]. Delivering functional cells, such as stem cells, directly to the bone trauma site can promote bone regeneration through osteogenic differentiation and the secretion of relevant growth factors [72]. SAP hydrogels have become an important stem cell carrier due to their excellent biocompatibility and facilitation of cell adhesion, migration, and culture [73]. BD PuraMatrix Peptide Hydrogel is a 3D cell culture model derived from SAP RADA16 hydrogels and has been widely used in bone tissue repair. Schwann cells (SCs) were cultured using PuraMatrix gel loading before being transplanted into the rat spinal cord injury model [74]. In vivo experiments showed that the transplanted SCs could proliferate and migrate in the hydrogel and that the host SCs migrated to the hydrogel in large quantities, promoting good bone marrow repair. The functionalized peptide hydrogel is a novel material with great application prospects.

Researchers hope to explore whether BMSCs can differentiate into cartilage using in vitro chondrogenic cultures of SAP hydrogels containing BMSCs. An injectable SAP hydrogel encapsulating BMSCs has shown excellent cartilage regeneration [75,76]. A novel functionalized self-assembled peptide hydrogel (KLD12/KLD-12-LPP, KLPP) containing an LPP was created [77]. The KLPP hydrogel induced and enhanced the migration of chondrocytes and BMSCs in an in vitro 3D culture system [48,78]. When injected in vivo, the KLPP hydrogel supported the targeted migration of chondrocytes and BMSCs and accelerated cartilage repair. SAP hydrogels can be used as a platform for a biomimetic bone tissue microenvironment and for evaluating osteoclast differentiation and function. Therefore, an SAP gel system loaded with osteoclasts was created and coated with hydroxyapatite nanoparticles to realize the biomimetic microenvironment of the osteoclast-bone interface [79]. A new bone regeneration/resorption model could be created by loading an inhibitor of osteoclast differentiation into the SAP hydrogel [80,81].

HUVECs were cultured with a novel SAP hydrogel (RATEA16) scaffold containing vascular endothelial growth factor (VEGF) to investigate its effect in promoting the proliferation of human umbilical vein endothelial cells (HUVEC) and angiogenesis. The HUVECs encapsulated in the RATEA16 hydrogels were round and proliferated efficiently and continuously [82]. The RADA-16 peptide was synthesized using solid-phase peptides, and well-defined hydrogels were formed by peptide supramolecular self-assembly. TGF-β1 was then loaded into the RADA-16 hydrogel. The TGF-β1/RADA-16 hydrogel had good biological properties, significantly accelerated the chondrogenic differentiation of BMSCs, and decreased the production of pro-inflammatory factors. HUVECs cultured on VEGF-containing RATEA16 hydrogels for 24 h formed vascular-like structures [83].

The SAP hydrogel can be loaded with cells to promote bone tissue regeneration and osteogenic active factors to promote bone tissue repair. Incorporating osteogenic active agents into bone filler materials can accelerate bone tissue regeneration. SAP hydrogels can control the slow release of osteogenic active agents locally and are widely used to deliver active bone regeneration agents [84]. One study created RATEA16, an SAP hydrogel able to load the VEGF and bone-morphogenetic protein 2 (BMP2) [15]. Its results showed that the gel delivery system could deliver growth factors efficiently, ensure that the concentration of released VEGF/BMP2 was maintained in the active range, and promote the angiogenesis of HUVECs and the osteogenic capacity of stem cells. The SAP D-RADA16-hydroxyapatite hydrogel loaded with basic fibroblast growth factor (bFGF) can maintain the continuous and stable release of bFGF in the bone defect site, accelerating the bone’s healing rate [85,86]. One study designed self-assembled KLD hydrogels loaded with platelet-derived growth factor-BB and heparin-binding insulin-like growth factor-1 for cartilage injury repair [87]. The osteogenic peptide was chemically coupled to the SAP KLD to enhance the osteogenic activity of the SAP hydrogels [47]. Its results showed that the created self-assembled bioactive peptide hydrogels could enhance ALP activity, increase calcium deposition, promote human BMSC proliferation [88], and upregulate the expression of key osteogenic markers such as ALP, COL-1, osteoprotegerin (OP), and osteocalcin (OCN) [89,90,91].

### 3.2. SAP Hydrogels in Skin Rejuvenation

SAPs have important applications in repairing traumatized skin, where they can be used as scaffolds for skin cells and reservoirs for active compounds to accelerate wound healing [92]. The RADA16-I, KLT, and RGD nanopeptides can self-assemble into hydrogels [93]. Human umbilical cord mesenchymal stem cell (hUC-MSC) spheroids were combined with SAP hydrogels for transplantation into a mouse model of diabetic skin trauma [94]. The hUC-MSC SAP hydrogel had better anti-inflammatory and pro-angiogenic effects than traditional stem cell transplantation, giving it better clinical application value. Another study reported that an SAP (KGH) hydrogel had an enhanced effect on ECM remodeling and angiogenesis in diabetic mice and, therefore, could play a role in accelerated chronic wound healing [39]. In vivo studies have shown that when injected, a KGH hydrogel accelerated wound healing while promoting angiogenesis, cell proliferation, and granulation tissue formation [95]. In vitro studies have also shown that a KGH hydrogel created a 3D microenvironment for skin cells, maintained the continuous growth of cell spheres, and increased the secretion of growth factors from skin keratinocytes [96]. Mechanistically, the KGH hydrogel may promote wound tissue regeneration by activating the Rho/ROCK and TGF-β/MEK/MAPK pathways.

Studies have explored the role of an SAP (SCIBIOIII) hydrogel with a unique nanofiber structure mimicking the natural ECM in chronic diabetic wound repair [97]. Their results showed that SCIBIOIII hydrogels had good biocompatibility in vitro and provided a 3D microenvironment for epidermal cells to grow sustainably. Mel-d1 was created by grafting SAPs onto o-carboxymethyl chitosan (O-CMCS), showing a compact spatial structure and good drug retardation performance, and incorporating a novel AMP based on bee venom proteins with equivalent antimicrobial activity and lower cytotoxicity than ciprofloxacin (CIP). In vivo tests showed that the O-CMCS/SAP hydrogels loaded with CIP and mel-d1 accelerated wound healing and skin tissue regeneration. The researchers found that compared to control groups (chitosan, poly[DL]lactic acid, COL-I, and a blank), RADA16-I improved the creation and disappearance of scars by 3–5 days and increased the rate of wound contraction by 20–30% without significant edema [98]. Immunohistochemical staining showed high RADA16-I expression in neoplastic tissues such as skin and glands. An SAP gel (RADA16/RADA16-sp) incorporating substance P was used to promote neovascularization and recruit intrinsic mesenchymal stem cells (MSCs) [99]. Their results showed that the human dermal fibroblasts (HDFs) with RADA16-sp poly(lactide-co-epsilon-caprolactone) (PLCL) scaffolds induced significantly more blood vessels and MSCs than the other groups. Therefore, their results indicated that HDFs with RADA16-sp PLCL scaffolds could induce vascularization and MSCs in vitro. The composite hydrogel was used to construct a new concept with high clinical application value. By investigating the role of SAP hydrogels in supporting skin-derived precursors (SKPs) in hair follicle neogenesis, researchers found that hydrogels formed by combining RADA16 and RGD-containing PRGs promoted SKP proliferation [58]. High SKP survival and the high expression of hair-induced marker genes such as Alp and bone morphogenetic protein 6 (Bmp6) significantly improved hair regeneration in mice.

### 3.3. SAP Hydrogels in Nerve Regeneration

Regeneration of neuronal tissue and its repair is complex since nerve cells are incapable of regeneration or have a very weak regenerative capacity. SAP hydrogels can repair peripheral nerve injury by promoting cell proliferation or the slow release of loaded drugs [100]. When a nerve is traumatically injured, it is difficult for nerve cells to differentiate and regenerate in vivo [101]. The poor regeneration of neural tissue can lead to permanent brain damage after traumatic brain injury or neurodegenerative lesions. However, the emergence of histological engineering has brought therapeutic strategies for neurological injuries [102]. Neural stem/progenitor cells (hNS/PC), human adipose-derived MSCs (hADSCs), and human meningioma stem cells have the potential to proliferate and differentiate into neurons and glial cells. The implantation of human meningioma stem cells into a rat model of acute traumatic brain injury and the implantation of nanofiber scaffolds containing human meningioma stem cells into an animal model of traumatic brain injury reduced the inflammatory reaction process and reactivated gliosis at the injury site, decreased apoptosis, and markedly improved the recovery of brain function [103,104]. Brain-derived hNS/PCs and hADSCs from patients with epilepsy were implanted into PuraMatrix hydrogel. Experimental studies in rats showed that these hydrogels could improve functional recovery, reduce the lesion’s extent, decrease the inflammatory response, and reduce gliosis in the lesion area, providing potential cellular therapy for neurological disorders such as refractory epilepsy [105].

After spinal cord injury, a fluid-filled sac develops, and there is an inflammatory response and gliosis at the injury site that limits nerve regeneration [106]. However, combining the structural microenvironment of peptide nanofibers with charge-induced action and adding growth factors, enabling their sustainable release, created an artificial biological microenvironment that triggered axonal regeneration and modulated local inflammatory responses [107,108]. In vivo and in vitro experiments showed that transplantation of microvascular cells into the SAP RADA16-I hydrogel resulted in the formation of a tightly connected blood–spinal barrier inside the peptide scaffold. In addition, a higher density of axons was seen at the graft or injury site, showing potential therapeutic value for spinal cord injuries [109]. In vitro studies found that the SAP hydrogel SPG-178 promoted the growth of motor neuron protrusions and increased the expression of nerve growth factor (NGF), brain-derived neurotrophic factor (BDNF), neurotrophin 4 (NTF4/NT-4), and neurotrophic receptor tyrosine kinases 1 (NTRK1/TRKA) and 2 (NTRK2/TRKB). In vivo studies showed that SPG-178 could reduce the inflammatory response and glial scar formation, providing new evidence that it acts as a scaffold to treat the spinal cord by inducing neuroprotective factors [110].

**Figure 2:**
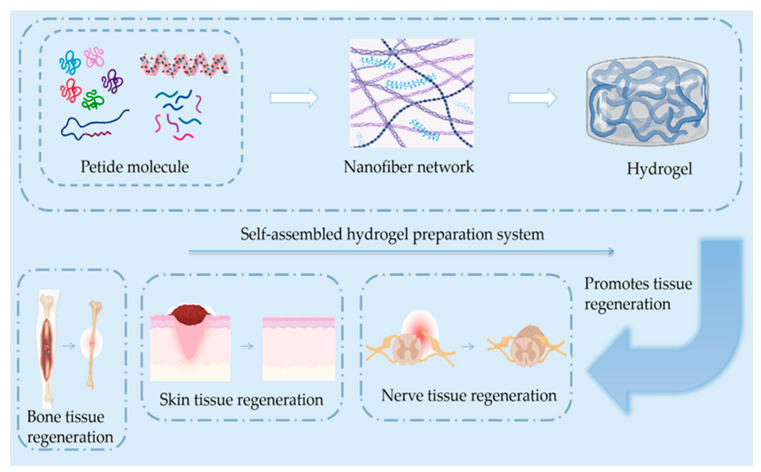
Simple schematics of the composition of the self-assembled hydrogel and skin, bone, and nerve regeneration. The regeneration process includes anti-inflammatory and hemostatic blood vessels and new scar tissue generation.

One study evaluated the effects of various scaffolds on cell adhesion, proliferation, and expression of functional genes using rat stem cells, finding that nanocomposite structures of peptide co-electrospun scaffolds with cellular recognition function could effectively improve the cells’ proliferation ability, accelerating the growth of axons and promoting the repair of functional nerves to treat peripheral nerve defects [62]. RAD-RGI SAP hydrogels were prepared using RADA16-I as a backbone and adding a BDNF-based neurotrophic peptide at its C-terminus. The functionalized peptide RAD/RGI hydrogel was found to provide a suitable microenvironment for axonal regeneration and glial cell growth and a synergistic effect in accelerating the repair of peripheral nerves in a rat sciatic nerve injury model [101,111]. Researchers designed a panel of eight mitochondria-derived peptides (MDPs) and successfully self-assembled them into injectable nanofiber hydrogels [67,112]. Their in vivo experiments demonstrated that MDP hydrogels injected into the sciatic nerve injury area in rats promoted macrophage recruitment to the injury area and degraded efficiently over time. This finding suggested that MDPs could promote the growth of neural protrusions after peripheral nerve injury and multicellular repair (Figure 2 and Table 3).

## 4. Conclusions and Outlook

Self-assembled hydrogels have attracted attention in regenerative medicine due to their biocompatibility, injectability, low immunogenicity, and flexibility in loading cells, proteins, and small molecule drugs [17]. However, since tissue repair is a complex process affected by many factors, self-assembled hydrogels could be loaded with various stem cells and active factors simultaneously to achieve synergistic effects of multiple strategies [113]. SAP hydrogels have shown the following characteristics during their application in tissue engineering: (1) they contain natural amino acids and are a nanostructured material; (2) when dissolved in sterile ionized water, the resulting hydrogel can be injected and is thoroughly degraded by proteolytic enzymes, greatly reducing the rejection of foreign substances; (3) SAPs are good biological materials with a better biocompatibility and better 3D structure for cell survival than other traditional materials; and (4) SAP fragments can be turned into desired nanofiber functional materials.

SAP hydrogels have good functional support for tissue defects but are less than ideal for extensive defects and/or load-bearing bone defects. When treating large bone defects, SAPs can be functionalized and combined with 3D printing and other technologies to mimic better the bone tissue’s regenerative microenvironment, which is rich in stem cells and activating factors, while being cultured in hydrogels before transplantation to improve regeneration [114]. In conclusion, the future development of SAP tissue-engineered repair hydrogels should focus on constructing biomimetic gels using multiple strategies, improving hydrogel mechanical strength, and controlling the gel degradation rate. With the continuous advancement of tissue engineering and cross-fertilization of multiple technologies, SAP hydrogels are expected to provide increasing clinical treatment options for bone defects, skin wounds, and nerve repair. This technology has resulted in a series of breakthroughs at the animal and cellular levels and has important applications in tissue repair.

While tissue engineering is developing rapidly, many problems remain, such as the mechanization of hydrogels with 3D structures being unstable. In addition, we need to explore how to set up a comprehensive monitoring system for this new biomaterial. Moreover, we need to investigate integrating self-assembled hydrogels with other materials or drugs to provide new therapeutic approaches to control their repair of damaged targets in tissue regeneration. For example, we need to examine whether it is possible to add other peptides with different functions of small polypeptide fragments and/or growth factors to a major repair bone regeneration peptide to give it osteogenic and other properties. Consequently, the resulting composite would have controllable biotargeting characteristics when applied in tissue regeneration. These difficulties and challenges will push forward developments in this field.

## Figures and Tables

**Table 1:** Classification of self-assembled peptide (SAP) hydrogels and other features.

Categorizations	Preparation Methods	Typology	Influencing Factors	Applications	Reference
Self-assembled short peptide hydrogels	1. Enzyme-catalyzed gel formation method 2. Self-assembly method 3. Crosslinking method 4. Solution mixing method	1. β-folded short peptide 2. Short peptides based on catalytic reactions	1. pH value 2. Peptide concentration 3. Temperatures 4. Ionic concentration 5. Chirality	1. Three dimensional tissue cell culture of various tissue cells and various stem cells 2. Regenerative medicine and tissue engineering 3. Three dimensional tissue printing 4. Continuous release of small molecules, growth factors, and monoclonal antibodies 5. Accelerates wound healing in skin and diabetic ulcers 6. Drug delivery and antitumor therapy. 7. Antimicrobial and wound repair	[19,20,21,22,23,24,25]
Self-assembling peptide hydrogels	1. Peptide molecules assemble themselves to form hydrogels 2. Preparation of hydrogels by self-assembly of peptides assisted by foreign aid molecules	1. Ion complementary peptides 2. Surfactant-based peptides 3. Chemical group-modified peptides			

**Table 2:** Composition of SASP hydrogels and their advantages.

Materials	Preparation Process	Functionalities	Type of Drug Load	Advantages/Disadvantages	Reference
RADA16, Amps	Direct coupling	Accelerates epithelial production, neovascularization, and collagen fiber production, significantly accelerating wound healing.	MGF E-peptide	The hydrogel prepared in this way is heat-sensitive, which facilitates drug release for wound healing.	[17]
TA, siRNA, PVA, HLC,	Direct coupling	Accelerated healing of diabetic wounds by promoting macrophage polarization, collagen production, and angiogenesis.	siRNA	The hydrogel has hemostatic, anti-inflammatory, antimicrobial, and pre-cellular proliferative properties and has demonstrated therapeutic efficacy throughout the stages of acute wound healing.	[38]
KGH, Ac-KLDLKLDLKLDLGGH-CONH2; KLD12, AcKLDLKLDLKLDL-CONH2	Direct coupling	Enhanced extracellular matrix (ECM) remodeling and angiogenesis to promote chronic wound healing in diabetic mice.	KGH	SAP hydrogel promotes secretion of ECM proteins and gf in skin cells through activation of Rho/ROCK and TGF-ß/MEK/MAPK pathways.	[39]
KLD	Direct coupling	Expression levels of osteogenic markers, collagen type I (COL-1), osteoblastin (OP), and osteocalcin (cyanate) were also significantly increased.	hMSCs	This hydrogel increased the injectable properties as well as facilitated the osteogenesis of hMSCs upon entry into the organism.	[47]
KLD-12/KLD-12-LPP, KLPP	Direct coupling	Promotes simultaneous recruitment of endogenous chondrocytes and bone marrow mesenchymal stem cells, interfacial integration, and cartilage regeneration. Promotes simultaneous recruitment of endogenous chondrocytes and bone marrow mesenchymal stem cells, interfacial integration, and cartilage regeneration.	LPP	KLPP hydrogel is less cytotoxic and significantly induces chondrocyte migration and increases BMSC migration.	[48]
Linked laminin-derived IKVAV, RADA16	Direct coupling	Inducing adhesion of encapsulated neural stem cells (NSCs) and consequent differentiation towards neurons	Linked laminin-derived IKVAV	This hydrogel formed a structure similar to brain tissue, improved the survival of encapsulated NSCs, and also reduced the formation of glial astrocytes.	[51]

**Table 3:** Materials and applications of SAP hydrogels.

Materials	Region of Action	Treatments for Diseases	Vantage	Reference
KLD12/KLD-12-LPP	Chondrocytes and bone marrow mesenchymal stem cells	Cartilage regeneration	Chondrocytes and BMSCs support directional migration and can accelerate cartilage repair	[77]
RATEA16, VEGF	Human umbilical vein endothelial cells	Cartilage regeneration	Promotion of angiogenesis	[82]
TGF-β1/RADA-16	Bone marrow mesenchymal stem cells (MSC)	Regeneration of bone marrow	Enhances chondrogenic differentiation and reduces the production of pro-inflammatory factors	[83]
RADA16-I, KLT and RGD nanopeptides	HUC-MSCs globules	Diabetic skin trauma	Better anti-inflammatory and pro-angiogenic effects	[94]
Microvascular cells, RADA16-I	Inside the peptide scaffold	Spinal cord injury	Formation of a tightly connected blood–spinal barrier	[109]
RADA16-I, neurotrophic peptide (RAD-RGI)	Rat sciatic nerve injury model	Sciatic nerve injury	Accelerated peripheral nerve repair	[111]
VEGF/ BMP-2, RATEA16	Human umbilical vein endothelial cells, stem cells	Bone defects	Promoting angiogenesis in human umbilical vein endothelial cells and osteogenic capacity of stem cells	[15]
RADA16, RGD, PRG	Skin-derived precursors (SKPs)	Follicle regeneration	Increased SKP survival and gene expression of Akp2, Bmp6, and others	[58]

## Data Availability

Not applicable.
